# Supplementary material for: Structural and functional neuroimaging of hippocampus to study adult neurogenesis in long COVID-19 patients with neuropsychiatric symptoms: a scoping review
Source: PeerJ. 2025 Jun 27;13:e19575. doi: 10.7717/peerj.19575 (PMC12208109; doi:10.7717/peerj.19575)
Supplement: Supplemental Information 1 [file peerj-13-19575-s001.docx]

**Table S1. The adjusted search terms as per searched electronic databases**

| Database | No | Search Query | Results |
| --- | --- | --- | --- |
| PubMed | | | |
|  | #1 | "Neurogenesis"[MeSH Terms] OR "Hippocampus"[MeSH Terms] OR "Lateral Ventricles"[MeSH Terms] | 1,44,236 |
|  | #2 | "adult neurogenesis"[Title/Abstract] OR "hippocampal neurogenesis"[Title/Abstract] OR "olfactory neurogenesis"[Title/Abstract] | 7,609 |
|  | #3 | "Neurogenesis"[MeSH Terms] OR "Hippocampus"[MeSH Terms] OR "Lateral Ventricles"[MeSH Terms] OR "adult neurogenesis"[Title/Abstract] OR "hippocampal neurogenesis"[Title/Abstract] OR "olfactory neurogenesis"[Title/Abstract] | 1,46,489 |
|  | #4 | "Post-Acute COVID-19 Syndrome"[MeSH Terms] OR "long covid 19"[Title/Abstract] OR "COVID-19"[Title/Abstract] OR "SARS-CoV-2"[Title/Abstract] | 3,62,199 |
|  | #5 | #3 AND #4 | 49 |
| Scopus | | | |
|  | #1 | TITLE-ABS-KEY ( ( "Neurogenesis" OR "Hippocampus" OR "Lateral Ventricles" OR "adult neurogenesis" OR "hippocampal neurogenesis" OR "olfactory neurogenesis" ) ) | 274,220 |
|  | #2 | TITLE-ABS-KEY ( ( "Post-Acute COVID-19 Syndrome" OR "long covid 19" OR "COVID-19" OR "SARS-CoV-2" ) ) | 533,108 |
|  | #3 | #1 AND #2 | 312 |
| EMBASE | | | |
|  | #1 | 'neurogenesis':ti,ab,kw OR 'hippocampus':ti,ab,kw OR 'lateral ventricles':ti,ab,kw OR 'adult neurogenesis':ti,ab,kw OR 'hippocampal neurogenesis':ti,ab,kw OR 'olfactory neurogenesis':ti,ab,kw | 206,348 |
|  | #2 | 'post-acute covid-19 syndrome':ti,ab,kw OR 'long covid 19':ti,ab,kw OR 'covid-19':ti,ab,kw OR 'sars-cov-2':ti,ab,kw | 402,979 |
|  | #3 | #1 AND #2 | 187 |
| Web of Science | | | |
|  | #1 | TS=(("Neurogenesis" OR "Hippocampus" OR "Lateral Ventricles" OR "adult neurogenesis" OR "hippocampal neurogenesis" OR "olfactory neurogenesis")) | 188,499 |
|  | #2 | TS=(("Post-Acute COVID-19 Syndrome" OR "long covid 19" OR "COVID-19" OR "SARS-CoV-2")) | 315,962 |
|  | #3 | #1 AND #2 | 159 |
| Cochrane | | | |
|  | #1 | MeSH descriptor: [Neurogenesis] explode all trees | 22 |
|  | #2 | (adult NEXT neurogenesis):ti,ab,kw OR (hippocampal NEXT neurogenesis):ti,ab,kw OR (olfactory NEXT neurogenesis):ti,ab,kw | 44 |
|  | #3 | #1 OR #2 | 63 |
|  | #4 | MeSH descriptor: [Post-Acute COVID-19 Syndrome] explode all trees | 59 |
|  | #5 | (Post-Acute COVID-19 Syndrome):ti,ab,kw OR (COVID-19):ti,ab,kw OR (SARS-CoV-2):ti,ab,kw | 16993 |
|  | #6 | #4 OR #5 | 16993 |
|  | #7 | #3 AND #6 | 0 |
