# Supplementary material for: Structural and functional neuroimaging of hippocampus to study adult neurogenesis in long COVID-19 patients with neuropsychiatric symptoms: a scoping review
Source: PeerJ. 2025 Jun 27;13:e19575. doi: 10.7717/peerj.19575 (PMC12208109; doi:10.7717/peerj.19575)
Supplement: Supplemental Information 2 [file peerj-13-19575-s002.docx]

Table S2: Participant and study characteristics

| Author., et al (Year) | Type of study | Clinical type of COVID-19 | | | | | | | | Mean days of hospitalization | | | | | | H/oCOVID-19 vaccination | dpi -scan | | H/o Psychiatric illness | | | Comorbidities | | | | | | | | | | |  |
| --- | --- | --- | --- | --- | --- | --- | --- | --- | --- | --- | --- | --- | --- | --- | --- | --- | --- | --- | --- | --- | --- | --- | --- | --- | --- | --- | --- | --- | --- | --- | --- | --- | --- |
|  |  | Mild | moderate | severe | | | critical | | Severity of COVID-19 |  |  | | |  |  | | |  | |  | Hypertension | | Diabetes | | Dyslipedemia | | | Cardiovascular disorder | | Seizure disorder | | Respiratory disordr |  |
|  |  | n(%) | n(%) | n(%) | | | n(%) | | WHO score | mean | SD | | | Hospitalization % |  | | |  | |  |  | |  | |  | | |  | |  | |  |  |
| Besteher et al., 2022 | Case control | 26 | 4 |  | | |  | | 2.45 (±1.06) |  |  | | |  | not mentioned | | | 2-16 months (8.65) | | Nil |  | |  | |  | | |  | |  | |  |  |
| Cattarinussi et al., 2022 | Case control |  |  |  | | |  | | Not reported |  |  | | | 28 (38.4%) | not mentioned | | | 132 ± 67 days | | Nil |  | |  | |  | | |  | |  | |  |  |
| Du et al., 2021 | retrospective observational cohort study | 10 |  | 9 | | |  | |  | 19.16 | 10.383 | | |  | not mentioned | | | 345.790±15.796 days | | Nil |  | |  | |  | | |  | |  | |  |  |
| Lu et al., 2020 | Case control | 47 (78.33%) |  | 12 (20.00%) | | | 1 (1.67%) | |  | 15.35 | 6.05 | | |  | not mentioned | | | 97.46 ± 8.01 | |  | 13 (21.67%) | | 6 (10.00%) | | | | |  | |  | |  |  |
| Taskiran-Sag et al., 2023 | Retrospective Case control |  |  |  | | |  | | Non severe |  |  | | |  | not mentioned | | | 2 weeks after 16 to 120 days (median 112 days) of recovery from COVID | |  |  | |  | |  | | |  | |  | |  |  |
| Ergül et al., 2022 | Case control |  |  |  | | |  | | Not reported |  |  | | |  | not mentioned | | | 1 to 3 weeks after recovery | |  |  | |  | |  | | |  | |  | |  |  |
| Esposito et al., 2021 | Case control | 20 | 1 | 6 | | | 0 | |  |  |  | | |  | not mentioned | | | 13.0 ± 7.4 day (1-21) after the period of infection (10 and 76 days(31.8 ± 21.0 days)). | | nil |  | |  | |  | | |  | |  | |  |  |
| Yousefi-Koma et al., 2021 | case report |  |  |  | | |  | | Not reported |  |  | | |  | not mentioned | | | 6 months | |  |  | |  | |  | | |  | |  | |  |  |
| Rothstein, 2023 | cohort study | 24 (100%) |  |  | | |  | |  | 0 | 0 | | | 0 | not mentioned | | | 85 days | | 29.1% depression or anxiety |  | | 12.50% | |  | | |  | | 4.10% | |  |  |
| Balsak et al., 2023 | Retrospective Case control | 46 | | | 28 | 0 | |  | |  | |  | 28/74 | | | not mentioned | 3-6 months | | nil | | | nil | | nil | |  | nil | | nil | | nil | | |
| Tu et al., 2021 | longitudinal case control | 117 (93%) | 0 | 9 (7%) | | | 0 | |  | 12.6 | 5 | | |  | not mentioned | | | 5-6 months | |  | 29 (23%) | | 8 (6%) | |  | | |  | |  | |  |  |
| Díez-Cirarda et al., 2023 | Case control |  |  |  | | |  | | not reported. 33% hospitalised |  |  | | | 28 (33%) | not mentioned | | | 11.08 ± 4.47 months. | | Nil | 20 (23.80%) | | 9 (10.71%) | | 22 (26.19%) | | | | |  | |  |  |
| Wingrove et al., 2023 | an exploratory, observational study | 39 (100%) | 0 | 0 | | | 0 | |  | 0 | 0 | | | 0 | not mentioned | | | 168±43 days | |  |  | |  | |  | | |  | |  | |  |  |
| Franke et al., 2023 | cohort |  |  |  | | |  | | Not reported |  |  | | |  | None were vaccinated | | | >3 months | | Nil | nil | | nil | | nil | | | nil | | nil | | nil |  |
| Mucciolia et al., 2023 | Case control | 20 (80%) |  | 2 (8.7%) | | | 1 (4.3%) | |  |  |  | | | 3(13%) | not mentioned | | | 11 ±5 months (2-19 months) | |  |  | |  | |  | | |  | |  | |  |  |
| CARROLL ET AL 2020 | case report |  |  |  | | | 1 (100%) | |  |  |  | | |  | not mentioned | | | 3 months before, 56th day and 78th day after infection | | Nil |  | | 1 (100%) | |  | | |  | |  | |  |  |
| Barnden et al., 2023 | Case control |  | 10 | | | |  | | moderate or worse severity |  |  | | |  | not mentioned | | | WHO- onset of symptoms less than 3 months post infection and persisting for atleast 3 months | | Nil | nil | | nil | | nil | | | nil | | nil | | nil |  |

Table S3: Neuropsychiatric symptoms studied in the included studies

| Author., et al (Year) | Neuropsychiatric symptoms | | | | | | | | | | | | | | | | | | | | | | | | | | | | | | | | Observation |
| --- | --- | --- | --- | --- | --- | --- | --- | --- | --- | --- | --- | --- | --- | --- | --- | --- | --- | --- | --- | --- | --- | --- | --- | --- | --- | --- | --- | --- | --- | --- | --- | --- | --- |
|  | Headache |  | Speech impairment |  | Vision change |  | hearing loss |  | Anosmia/hyposmia/dysosmia |  | Ageusia/hypogeusia/dysgeusia |  | Myalgia |  | Decreased appetite |  | Fatigue |  | Depressed mood |  | Anxiety |  | Mood changes |  | Cognitive impairment/brain fog |  | sleep disturbances |  | Memory impairment |  | difficulty in concentration |  |  |
|  | Yes/No | % | Yes/No | % | Yes/No | % | Yes/No | % | Yes/No | % | Yes/No | % | Yes/No | % | Yes/No | % | Yes/No | % | Yes/No | % | Yes/No | % | Yes/No | % | Yes/No | % | Yes/No | % | Yes/No | % | Yes/No | % |  |
| Besteher et al., 2022 |  |  |  |  |  |  |  |  |  |  |  |  |  |  |  |  | Yes |  | Yes |  |  |  |  |  |  |  |  |  | Yes |  | Yes |  |  |
| Cattarinussi et al., 2022 |  |  |  |  |  |  |  |  |  |  |  |  |  |  |  |  |  |  |  |  |  |  |  |  |  |  |  |  |  |  |  |  |  |
| Du et al., 2021 | Yes | 26.3 |  |  |  |  |  |  | Yes | 5.6 | Yes | 5.6 | Yes | 26.3 | yes | 5.6 | Yes | 21.1 |  |  |  |  |  |  |  |  | yes |  |  |  |  |  | LC-Fatigue and myalgia persistent in 1 year and headach increased |
| Lu et al., 2020 | yes | 10.0 |  |  | yes | 1.7 | yes | 1.67 | yes | 3.3 | yes | 1.7 | yes | 25.0 |  |  | yes | 6.7 |  |  |  |  | yes | 16.7 |  |  |  |  | yes | 28.3 |  |  |  |
| Taskiran-Sag et al., 2023 | yes |  |  |  |  |  |  |  | yes |  | yes |  |  |  |  |  |  |  |  |  | yes |  |  |  | yes |  | yes |  |  |  |  |  |  |
| Ergül et al., 2022 |  |  |  |  |  |  |  |  | yes |  | yes |  |  |  |  |  |  |  |  |  |  |  |  |  |  |  |  |  |  |  |  |  | Olfactory disorder score and gustatory disorder scores were higher in LC [(1.55 ± 0.89) and (1.45 ± 1.19)] compared to HC [0]. - modified version of the validated Monell-Jefferson Taste–Smell questionnaire |
| Esposito et al., 2021 | no |  |  |  | no |  | no |  | yes | 100.0 | yes (hypo) | 22.2 |  |  |  |  |  |  |  |  |  |  |  |  | no |  |  |  | no |  |  |  | Duration of olfactory symptoms - 36.64± 27.5 (11-89 days) |
| Yousefi-Koma et al., 2021 |  |  |  |  |  |  |  |  | Yes | 100.0 |  |  |  |  |  |  |  |  |  |  |  |  |  |  |  |  |  |  |  |  |  |  | Hyposmia for 3 months |
| Rothstein, 2023 | yes | 41.6 | yes | 41.6 |  |  |  |  | yes | 29.1 | yes | 29.1 |  |  |  |  | yes | 87.5 |  |  |  |  |  |  | yes | 91.6 |  |  |  |  |  |  |  |
| Balsak et al., 2023 | yes | 100.0 |  |  |  |  |  |  |  |  |  |  |  |  |  |  |  |  |  |  |  |  |  |  |  |  |  |  |  |  |  |  | COVID-19 patients with headache were recruited |
| Tu et al., 2021 |  |  |  |  |  |  |  |  |  |  |  |  |  |  |  |  |  |  |  |  |  |  |  |  |  |  |  |  |  |  |  |  |  |
| Díez-Cirarda et al., 2023 |  |  |  |  |  |  |  |  | yes | 69.0 |  |  |  |  |  |  | yes | 81.0 | yes | 25 |  |  |  |  |  |  | yes | 82.1 |  |  |  |  |  |
| Wingrove et al., 2023 |  |  |  |  |  |  |  |  | yes | 20.5 |  |  |  |  |  |  |  |  |  |  |  |  |  |  |  |  |  |  |  |  |  |  | The study group includes 10 COVID-19 patients of age 50.79 years whose anosmia was recovered and 10 young COVID-19 patients of mean age 27.7 years with recovered anosmia in 4-6 weeks |
| Franke et al., 2023 |  |  |  |  |  |  |  |  |  |  |  |  |  |  |  |  |  |  |  |  |  |  |  |  | yes | 100.0 |  |  |  |  |  |  | Self reported symptoms |
| Mucciolia et al., 2023 |  |  |  |  |  |  |  |  | yes | 100.0 | yes | 39.1 |  |  |  |  | yes | 73.9 | Yes | 30 | Yes | 13 |  |  | yes | 4.3 |  |  | Yes |  |  |  | olfactory defect associated with dysosmia in 10 (43 %) cases (cacosmia, n =7, phantosmia, n =1, parosmia, n =3) and persistent gustatory dysfunction in 19 (83 %). |
| CARROLL ET AL 2020 |  |  |  |  |  |  |  |  |  |  |  |  |  |  |  |  |  |  |  |  |  |  |  |  |  |  |  |  | yes | 100 |  |  | History of forgetfulness |
| Barnden et al., 2023 | yes | 80.0 |  |  | yes | 50.0 |  |  | yes | 20.0 | yes | 20.0 |  |  |  |  | yes | 100.0 |  |  |  |  |  |  |  |  | yes | 100 | yes | 30 | yes | 70 | All 10 subjects reported moderate to very severe fatigue, muscle aches and unrefreshing and/or disturbed sleep. |

Table S4: Psychometric analysis of the neuropsychiatric symptoms studied in the included studies

| Author., et al (Year) | Psychometric analysis | | | | | | | | | | | | | | | | | | | | | | | | | | | | | Findings |
| --- | --- | --- | --- | --- | --- | --- | --- | --- | --- | --- | --- | --- | --- | --- | --- | --- | --- | --- | --- | --- | --- | --- | --- | --- | --- | --- | --- | --- | --- | --- |
|  | Cognitive impairment | | | Insomnia | | | Fatigue | | | Depression | | | Anxiety | | |  |  |  | Stress | | | Olfactory disturbance | | | PTSS | | | | |  |
|  | Scale used | Study group | Control | Scale used | Study group | Control | Scale used | Study group | Control | Scale used | Study group | Control | Scale used | Study group | Control | Scale used | Study group | Control | Scale used | Study group | Control | Scale used | Study group | Control | Scale used | Study group-M | Study group-F | Control M | Control F |  |
| Besteher et al., 2022 | MoCA | 26.21 (2.54) | 28.05 (1.82) | |  |  |  |  |  | MADRS | 12.24 (8.46) | 2.55 (1.79) | STAI -state | 43 (5.21) | 41.89 (4.03) | STAI-trait | 43.41 (7.63) | 43. 53 (6.22) |  |  |  |  |  |  |  |  |  |  |  | Higher cognitive dysfunction and depression in LC pts than HC |
| Cattarinussi et al., 2022 |  |  |  |  |  |  | Multidimension Fatigue Inventory | 56.8 (15.3) | 59.3 (5.7) | PHQ 9 | 4.3 (4.2) | 2.4 (2.2) | GAD 7 | 3.4 (4.3) | 2.4 (2.2 |  |  |  |  |  |  |  |  |  |  |  |  |  |  | Higher scores of depression in study than HC |
| Du et al., 2021 |  |  |  | Athens Insomnia Scale | 8.320±4.534 | 5.560±3.001 | |  |  | Hospital Anxiety and Depression Scale-D | 3.740±4.954 | 4.160±3.520 | Hospital Anxiety and Depression Scale-A | 3.210±4.131 | 5.040±3.458 | |  |  |  |  |  |  |  |  |  |  |  |  |  | Higher AIS scores (sleep disruptions) in LC than HC |
| Lu et al., 2020 |  |  |  |  |  |  |  |  |  |  |  |  |  |  |  |  |  |  |  |  |  |  |  |  |  |  |  |  |  |  |
| Taskiran-Sag et al., 2023 |  |  |  |  |  |  |  |  |  |  |  |  |  |  |  |  |  |  |  |  |  |  |  |  |  |  |  |  |  |  |
| Ergül et al., 2022 |  |  |  |  |  |  |  |  |  |  |  |  |  |  |  |  |  |  |  |  |  |  |  |  |  |  |  |  |  |  |
| Esposito et al., 2021 | MoCA | >15.5 | >15.5 |  |  |  |  |  |  |  |  |  |  |  |  |  |  |  |  |  |  |  |  |  |  |  |  |  |  |  |
| Yousefi-Koma et al., 2021 |  |  |  |  |  |  |  |  |  |  |  |  |  |  |  |  |  |  |  |  |  |  |  |  |  |  |  |  |  |  |
| Rothstein, 2023 |  |  |  |  |  |  |  |  |  |  |  |  |  |  |  |  |  |  |  |  |  |  |  |  |  |  |  |  |  |  |
| Balsak et al., 2023 |  |  |  |  |  |  |  |  |  |  |  |  |  |  |  |  |  |  |  |  |  |  |  |  |  |  |  |  |  |  |
| Tu et al., 2021 |  |  |  |  |  |  |  |  |  | PHQ-9 | m-4.9±5.1, f-6.5±5.1 | m-1.9±2.6, f-3.7±3.6 | GAD-7 | m - 4±4.5, f-5.8±4.6 | m-1.6±2.3, f-3.4±3.1 | |  |  |  |  |  |  |  |  | Posttraumatic-Stress Disorder (PTSD) Checklist for DSM-5 | 10.4 ± 13.1 | 12.5 ± 11.3 | 3.1±3.4 | 7.1±7.8 | Higher PCL-5 scores in LC than HC, higher PCL-5 scores in LC females than LC males, Increase in PCL-5 scores in 3-6 months when compared to post discharge |
| Díez-Cirarda et al., 2023 | Yes |  |  | PSQI | 9.61 ± 4.70 | Not reported | Modified Fatigue Impact Scale (MFIS) | 53.27 ± 14.97 | Not reported | Beck Depression Inventory-II | 14.39 ± 9.04 | Not reported | |  |  |  |  |  |  |  |  | Brief Smell Identification Test (BSIT) | 9.18 ± 2.34 | Not reported | |  |  |  |  | significant and positive correlations between CCL11 andMOG with cognition, and significant and negative correlations between GFAP and NfL with cognition. 2.Impairment in attention (42.9%; 36/84),memory (40.5%; 34/84), executive functions (38.1%; 32/84), but also in visuospatial ability (31%; 26/84), pro-cessing speed (28.6%; 24/84), and language (19%; 16/84) |
| Wingrove et al., 2023 |  |  |  |  |  |  |  |  |  |  |  |  |  |  |  |  |  |  |  |  |  | University of Pennsylvania Smell Identification Test (UPSIT | 15.63 ± 5.85 | Not reported | |  |  |  |  | UPSIT score in COVID-19 patients with recoverd anosmia (10) was 34.10 ± 1.85 and in young COVID-19 patients with recovered anosmia was 34.23 ± 2.00 |
| Franke et al., 2023 | MoCA | 32- subjective cognitive decline MoCA > 25(median 27, [26–29 points]), 18- pathological MoCA score ≤25 points (median 23, [13–25]) | | | | | | | | | | | | |  |  |  |  |  |  |  |  |  |  |  |  |  |  |  | 32 with subjective cognitive decline and 18 with pathological MOCA scores |
| Mucciolia et al., 2023 | MoCA | 25.74 | 2.51 |  |  |  | Modified Fatigue Impact Scale. | 35±20.12 |  | DASS21 | 5.65±4.82 |  | DASS21 | 5.09±3.42 |  |  |  |  | DASS21 | 8.22±4.88 |  |  |  |  |  |  |  |  |  | one-third of the patients had a pathological depression score, while three-fourth of patients had a pathological fatigue score |
| CARROLL ET AL 2020 |  |  |  |  |  |  |  |  |  |  |  |  |  |  |  |  |  |  |  |  |  |  |  |  |  |  |  |  |  |  |
| Barnden et al., 2023 | Stroop task | 0.162 | 0.139 |  |  |  |  |  |  |  |  |  |  |  |  |  |  |  |  |  |  |  |  |  |  |  |  |  |  | lower accuracy and slower reaction times in LC than HC. |

Table S5:

| Author., et al (Year) | Indirect immunofluorescence | | | Neuroimaging modalities | | | | | | |
| --- | --- | --- | --- | --- | --- | --- | --- | --- | --- | --- |
|  | Animal model | findings | Correlation with other factors | structural MRI | | | | | fMRI | |
|  |  |  |  |  | VBM | CBV | CBF | DTI | Activity | Resting |
| Besteher et al., 2022 |  |  |  | Yes | Yes |  |  |  |  |  |
| Cattarinussi et al., 2022 |  |  |  | Yes | Yes |  |  |  |  | Yes |
| Du et al., 2021 |  |  |  |  |  |  |  |  |  | Yes |
| Lu et al., 2020 |  |  |  | Yes | Yes |  |  | yes |  |  |
| Taskiran-Sag et al., 2023 |  |  |  | yes | yes |  |  |  |  |  |
| Ergül et al., 2022 |  |  |  | yes | yes |  |  |  |  |  |
| Esposito et al., 2021 |  |  |  | Yes | Yes |  |  | yes |  | yes |
| Yousefi-Koma et al., 2021 |  |  |  | Yes | Yes |  |  |  |  |  |
| Rothstein, 2023 |  |  |  | Yes | Yes |  |  |  |  |  |
| Balsak et al., 2023 |  |  |  |  |  |  |  | yes |  |  |
| Tu et al., 2021 |  |  |  | Yes | Yes |  |  |  |  | Yes |
| Díez-Cirarda et al., 2023 |  |  |  | Yes | Yes |  | yes |  |  | yes |
| Wingrove et al., 2023 |  |  |  | Yes | Yes |  | yes |  |  | Yes |
| Franke et al., 2023 | mouse | anti-neuronal autoantibodies were found in 52 % of all patients: n =9 in serum only, n =3 in CSF only and n =14 in both against myelin, Yo, Ma2/Ta, GAD65 and NMDA receptor, including cerebral vessel endothelium, Purkinje neurons, granule cells, axon initial segments, astrocytic proteins and neuropil of basal ganglia or hippocampus. | The patients with autoantibodies in CSF (with or without autoantibodies in serum) showed significantly lower MoCA scores compared to the PCS patients who also reported on predominant cognitive deficits but without detection of autoantibodies | yes |  |  |  |  |  |  |
| Mucciolia et al., 2023 |  |  |  | Yes | Yes |  |  |  |  | Yes |
| CARROLL ET AL 2020 |  |  |  | Yes |  |  |  |  |  |  |
| Barnden et al., 2023 |  |  |  |  |  |  |  |  | yes (Stroop task) | |
