# Supplementary material for: Structural and functional neuroimaging of hippocampus to study adult neurogenesis in long COVID-19 patients with neuropsychiatric symptoms: a scoping review
Source: PeerJ. 2025 Jun 27;13:e19575. doi: 10.7717/peerj.19575 (PMC12208109; doi:10.7717/peerj.19575)
Supplement: Supplemental Information 5 [file peerj-13-19575-s005.docx]

| Author., et al (Year) | Findings of neuroimaging study | | Correlation with symptoms | Correlation with other biomarkers | Mechanism reported (Yes/No) | Mechanism findings | Gaps reported in the study | Comments |
| --- | --- | --- | --- | --- | --- | --- | --- | --- |
|  | Findings | Relevant observation |  |  |  |  |  |  |
| Besteher et al., 2022 (Besteher et al. 2022) | Larger bilateral hippocampal GMV in LC | LC with NPS show brain alterations in GMV | Psychometric factors were not a predictor of GMV. Time since COVID-19 was an inverse predictor of hippocampal GMV | - | Yes | 1.Recovery due to AN and/or increased functional activity leading to hypertrophy of neurons and amplifications of dendritic connections. 2. Neuroinflammation | 1. Neural correlates for NPS burden in LC were not studied. 2 Neuroimaging findings were not correlated with severity of symptoms | Study conducted on long-COVID patients with neuropsychiatric symptoms such as fatigue, depression and cognitive deficits and a mean time since disease onset of 8 months (range 2–16 months) |
| Cattarinussi et al., 2022 (Cattarinussi et al. 2022) | Increase in intrinsic functional connectivity in the right hippocampus. | Functional abnormalities observed in hippocampus | Positive correlation between ReHo in the right hippocampus and severity of depression | - | Yes | COVID-19 causes changes in local FC in areas primarily involved in social behaviour and mood regulation, leading to the development of depressive symptoms, and these alterations possibly mediated by an inflammatory response. | 1. Time from the first positive molecular swab test for SARS-CoV-2 to the MRI scan acquisition varied greatly. 2.Whether the alterations in regional connectivity in hippocampus were due to COVID-19 or depression due to lockdown was not elucidated. 3. Psychopathological symptoms were retrospectively assessed, which may have led to recall biases 4. Not longitudinal in nature and no baseline data for comparison | 1. Only 44/79 (55.7%) of the study population had LC. 2.The study suggests that COVID-19 can result in changes in local FC in areas primarily involved in social behaviour and mood regulation, leading to the development of depressive symptoms, and these alterations may be mediated by an inflammatory response |
| Du et al., 2021 (Du et al. 2022) | Increased ALFF in hippocampus in LC when compared to HC with highest difference in left hippocampus | Increased brain activity in hippocampus in LC compared to HC |  | No correlation between ALFF in hippocampus and inflammatory biomarkers CRP, neutrophil and lymphocyte count | Yes | Compensatory repair of brain tissue following hypoxia or inflammation. | No comparison between acute COVID-19 and LC | 1. Brain regions at follow-up only showed higher ALFF values than controls; no regions exhibited lower ALFF values. 2. ALFF values were elevated in the left hemisphere, but not in the right hemisphere. |
| Lu et al., 2020 (Lu et al. 2020) | 1.Higher bilateral hippocampal GMV in LC compared to HC. 2 No difference in hippocampal MD, FA | Enlarged hippocampus in LC | 1. Negative correlation between bilateral hippocampal GMV and memory loss 2. Left hippocampal GMV negatively correlated with anosmia in acute COVID-19 but not in LC | 1. Left hippocampal GMV negatively correlated with WBC count | Yes | Neurogenesis and functional compensation | 1. Not enough patients with neurological dysfunction or olfactory loss were enrolled and therefore the relationship between GMV/diffusivity changes and olfactory symptoms could have been missed; 2) As a single-centred study, selection bias might result from limited ethnical and regional characteristics of the participants, and missed the effect of possible mutants of SARS-CoV-2 in other countries, which affects the generalization of the study results | 1. 33 (55.00%) patients had neurological symptoms during follow-up (scan) . Lower diffusivity parameters (MD, AD, RD) and higher FA values were recognized in the white matter from COVID-19 cohort. 2.All the diffusivity abnormalities in the white matter restricted in the right hemisphere, without asymmetrical symptoms reported by COVID-19 patients. |
| Taskiran-Sag et al., 2023 (Taskiran-Sag et al. 2023) | No difference in bilateral hippocampal thickness between LC and HC |  | Patients with anxiety had thicker right hippocampus compared to HC | No correlation between hippocampal thickness and inflammatory biomarkers CRP and neutrophil to lymphocyte ratio | No |  | 1. Lack of baseline data for comparison 2. Uncertainty in linking the neurological complaints to COVID-193. Self-reported nature of the symptoms |  |
| Ergül et al., 2022 (Ergül et al. 2022) | No difference in bilateral hippocampal volume between LC and HC |  | No correlation between hippocampal volume and olfactory disorder and gustatory disorder score | No correlation between hippocampal volume and biochemical parameters vitamin B12, Zn, Fe, ferritin, T4, TSH and endocannabinoids | No |  | Questionnaire method to evaluate olfactory and gustatory disturbances | 1. Twenty patients who recovered from COVID-19 (1 to 3 weeks since the patients got over the disease) but still express olfactory and gustatory complaints were included. 2. The duration of the acute stage of the infection not mentioned |
| Esposito et al., 2021 (Esposito et al. 2022) | Reduction in functional connectivity between hippocampus and insula in LC with olfactory disorder | Increased functional segregation in olfactory network in LC with higher olfactory loss |  |  | Yes | The olfactory network mimics a network of cognitive reserve, the olfactory loss configuring a sensory proxy of a characteristic COVID-19 reserve of neural plasticity | Lack of baseline data for comparison |  |
| Yousefi-Koma et al., 2021 (Yousefi-Koma et al. 2021) | Normal hippocampal volume and 12% reduction in the maximal standardized uptake value (SUVmax) in the left hippocampus | Functional impairment, not structural |  |  | Yes | Neuroinvasion by COVID-19 |  |  |
| Rothstein, 2023 (Rothstein 2023) | Larger hippocampal volume compared to computerised normalised volumes | Total brain volume was stable while cortical grey mater volume was diminished, and hippocampal volume was increased compared to computerised normalised volumes |  |  | Yes | Macroscopic volume changes associated with adult hippocampal neurogenesis | 1.longitudinal imaging studies to determine recovery. 2. No baseline imaging data for comparison | MRI results has been compared with sex and age matched healthy controls drawn from an existing normative database provided by the developer of NeuroQuant® (Cortechs Laboratories, San Diego, California) with values based on their percentage of intracranial volume. |
| Balsak et al., 2023 (Balsak et al. 2023) | No significant difference in FA of hippocampus between LC and HC and between inpatient, outpatient LC and HC |  |  | Negative correlation between FA from hippocampus and plasma LDH levels in LC patients who were hospitalized | Yes | Deterioration of axonal integrity and demyelination process secondary to hypoxia in hippocampus. | No baseline data for comparison |  |
| Tu et al., 2021 (Tu et al. 2021) | 1.Higher GMV in bilateral hippocampus in LC than HC. 2 Higher ALFF in bilateral hippocampus in LC when compared to HC | Larger GMVs and higher functional activities in bilateral hippocampus and amygdala, as compared to controls | Left hippocampal volume negatively correlated with PCL-5 scores |  | Yes | Enlarged structural volumes of the brain regions could be a functional compensation to cope with the acute stress and the ongoing COVID-19 related trauma | The study could not completely rule out the possible effects of COVID-19 infection and medications on abnormal brain structures. | The study was conducted in COVID-19 survivors. Though there was no mention of any persistent symptoms, the COVID-19 survivors' PTSS was analysed and its presence was not associated with any reason other than COVId-19 |
| Díez-Cirarda et al., 2023 (Díez-Cirarda et al. 2023) | Lower volume in all hippocampal subfields in LC when compared to HC except in CA3 body and parasubiculum subfields. 2. Higher intracellular volume fraction (FICVF)and higher orientation dispersion index (ODI) values in LC compared to HC 3. Lower hippocampal perfusion in LC compared to HC 4. Bilateral hippocampal volume was significantly and positively related to white matter volume adjacent to the left Para hippocampal and fusiform gyrus area 5. Reduced functional connectivity in PCS patients compared HC between the right head of the hippocampus and the left anterior Para hippocampal division and the parietal area, including supra-marginal and postcentral areas, overlapping the dorsal attention network | Hippocampal volume loss was accompanied by microstructural alterations, showing changes in the intracellular volume, together with hypoperfusion in LC | 1. Strong positive correlation between head hippocampal subfield volume and cognition 2. Reduced hippocampal perfusion was related to memory performance 3. Hippocampal volume differences were more accentuated in hospitalized patients compared to non-hospitalized patients in most of the hippocampal subfields. 4. Lower hippocampal perfusion values in hospitalized patients compared to non-hospitalized patients | Increased GFAP, MOG and Nfl in LC when compared to HC. 2. Positive correlation between GFAP and MOG and whole hippocampal volume 3. CCL11 (inhibitor of AN) showed negative and significant associations with dentate gyrus, CA3 head and CA4 head volumes of the hippocampus and NfL showed negative and significant associations with hippocampal head subfield | Yes | 1. Acute damage, such as hypoxia or acute neuro-inflammation 2. Consequence of persistent neuroinflammation and compensatory mechanism driven by astrocyte activation, and reduction of neurogenesis inhibition in the hippocampus 3. Unchaining neurodegenerative mechanisms. | 1. Cross sectional nature of the study 2. Biomarkers in the blood and not in CSF were studied |  |
| Wingrove et al., 2023 (Wingrove et al. 2023) | 1. Higher CBF in posterior hippocampus in LC with anosmia when compared to those COVID-19 patients with resolved anosmia 2. No difference in hippocampal gray matter density was observed between LC with anosmia, recovered group and HC. 3. No changes in global GM perfusion and no difference in CBF between COVID patients and HC |  | LC patients with anosmia has higher CBF in posterior hippocampus when compared to COVID-19 survivors whose anosmia was recovered in 4-6 weeks |  | Yes | 1. Subtle vascular effects that are only noticeable in the smaller diameter arteries supplying the brain (posterior cerebral artery) 2. Local changes in neuronal activity/metabolism, due to the generally accepted coupling between blood flow and metabolism | 1. No information on behavioural measures or comorbidities were collected 2. Longitudinal data not available | Only anosmia was considered in LC and other symptoms if any were not mentioned in the study |
| Franke et al., 2023 (Franke et al. 2023) | Nil | Cranial MR imaging did not reveal pathological findings correlating with cognitive impairment including atrophy. | none | None | yes | Humoral autoimmunity may contribute to the development of cognitive impairment in some PCS patients | 1. No comparison with HC 2. Smaller sample size |  |
| Mucciolia et al., 2023 (Muccioli et al. 2023) | 1. Bilateral hippocampal volume reduction in LC when compared to HC but the statistical significance was lost when corrected for multiple comparison 2. No differences in functional connectivity of hippocampus between LC and HC. 3. The olfactory network of patients with COVID-19- related olfactory dysfunction was overall less segregated into clusters of functionally associated components, which likely play specific functions in the central olfactory processing.4. Dysfunctional connectivity between right thalamus and right posterior hippocampus | No significant morphological and functional alterations in hippocampus were found in LC patients with olfactory dysfunction when compared with HC | None | - | Yes | lLss of sensory input due to anosmia, neuroinflammatory events, or neurodegeneration | Smaller sample size |  |
| Carroll et al 2020 (Muccioli et al. 2023) | 56th day - worsened right > left hippocampal atrophy compared to the MRI obtained 3 months prior to initial hospitalization, diffuse parenchymal volume loss, and no evidence of diffusion restriction. 78th day - no evidence of diffusion restriction or enhancement, but bilateral hippocampal atrophy had progressed | Worsening of hippocampal atrophy |  | Elevated systemic inflammatory markers, CRP | No |  |  | Postinfectious inflammation may have triggered refractory status epilepticus in a manner similar to the multisystemic inflammatory syndrome observed |
| Barnden et al., 2023 (Muccioli et al. 2023) | Weaker connectivity between bilateral hippocampus and the whole brain in LC when compared to HC |  | No correlation between hippocampal connectivity and clinical scores of heart rate variability, respiratory rate variability and Bell disability | - | Yes | Impaired cell membrane calcium transport and transient receptor potential melastatin 3 (TRPM3) reported dysfunction in LC | Pilot study with limited sample size and not longitudinal | Stroop task to investigate attention and concentration difficulties in LC with fatigue |
